# Spatio-Temporal Transformer with Kolmogorov–Arnold Network for Skeleton-Based Hand Gesture Recognition

**DOI:** 10.3390/s25030702

**Published:** 2025-01-24

**Authors:** Pengcheng Han, Xin He, Takafumi Matsumaru, Vibekananda Dutta

**Affiliations:** 1 Graduate School of Information, Production and System, Waseda University, Kitakyushu 808-0135, Japan; hexin@akane.waseda.jp (X.H.); matsumaru@waseda.jp (T.M.); 2 Institute of Micromechanics and Photonics, Faculty of Mechatronics, Warsaw University of Technology, 00-661 Warszawa, Poland; vibekananda.dutta@pw.edu.pl; 3 Waseda Institute of Advanced Study, Waseda University, Tokyo 169-8050, Japan

**Keywords:** hand gesture recognition, human–computer interaction (HCI), skeleton based, deep learning, graph convolutional networks, transformer, attention mechanism, feature extraction, continuous hand gesture recognition

## Abstract

Manually crafted features often suffer from being subjective, having an inadequate accuracy, or lacking in robustness in recognition. Meanwhile, existing deep learning methods often overlook the structural and dynamic characteristics of the human hand, failing to fully explore the contextual information of joints in both the spatial and temporal domains. To effectively capture dependencies between the hand joints that are not adjacent but may have potential connections, it is essential to learn long-term relationships. This study proposes a skeleton-based hand gesture recognition framework, the ST-KT, a spatio-temporal graph convolution network, and a transformer with the Kolmogorov–Arnold Network (KAN) model. It incorporates spatio-temporal graph convolution network (ST-GCN) modules and a spatio-temporal transformer module with KAN (KAN–Transformer). ST-GCN modules, which include a spatial graph convolution network (SGCN) and a temporal convolution network (TCN), extract primary features from skeleton sequences by leveraging the strength of graph convolutional networks in the spatio-temporal domain. A spatio-temporal position embedding method integrates node features, enriching representations by including node identities and temporal information. The transformer layer includes a spatial KAN–Transformer (S-KT) and a temporal KAN–Transformer (T-KT), which further extract joint features by learning edge weights and node embeddings, providing richer feature representations and the capability for nonlinear modeling. We evaluated the performance of our method on two challenging skeleton-based dynamic gesture datasets: our method achieved an accuracy of 97.5% on the SHREC’17 track dataset and 94.3% on the DHG-14/28 dataset. These results demonstrate that our proposed method, ST-KT, effectively captures dynamic skeleton changes and complex joint relationships.

## 1. Introduction

### 1.1. Background

Hand gesture recognition allows computers to capture and interpret human gestures through mathematical algorithms and execute commands based on these gestures to achieve human–computer interaction [1,2]. Hand gesture recognition has become a vibrant research field due to its wide application in various fields such as human–computer interaction, games [3], and non-verbal communication (including sign language interpretation [4]). In the field of human–computer interaction, hand gesture recognition is a very important component that enables natural and intuitive communication between users and machines without the use of traditional input devices such as mouse and keyboard. With the rapid development of this field, the technology has been applied to many fields, such as healthcare and industry, providing solutions for prosthetic control, rehabilitation assistance, medical assistive devices, industrial robots, and non-contact control operations of equipment. Among them, its role in supporting people with hearing impairment is particularly noteworthy. According to the World Health Organization (WHO) [5], more than 5% of the world’s population (about 430 million people) need rehabilitation treatment to cope with hearing loss. With population growth and aging, by 2050, it is expected that nearly 2.5 billion people will have varying degrees of hearing loss, of which at least 700 million will need rehabilitation. These shocking statistics highlight the urgent need for innovative assistive technologies. Facilitating the development of hearing assistive technologies and services, including sign language recognition and translation, can help bridge the communication gap and improve accessibility for this population.

Although vision-based hand gesture recognition has been extensively studied for decades, it still faces several challenges [6] that hinder its widespread application:

- **Lighting changes:** Under different lighting conditions, the color and outline of the hand change significantly, affecting the recognition accuracy.
- **Complex backgrounds:** In a complex background, the hand and background have similar colors or textures, making it difficult to accurately perform segmentation and identification.
- **Noise:** Image noise affects edge detection and feature extraction, reducing recognition accuracy.
- **Real time:** Many hand gesture recognition systems cannot meet real-time requirements, limiting their use in interactive applications.
- **Gesture diversity:** Gesture differences exist between cultures, regions, and individuals, requiring the establishment of more comprehensive databases and recognition models.

### 1.2. Motivation

Hand gesture recognition methods have evolved significantly with the continuous development of deep learning techniques. These methods can be broadly categorized into two main types: image-based methods and skeleton-based methods [7]. Image-based methods use RGB or RGB-D images, image sequences, or videos as input, relying on image-level features for hand gesture recognition. In contrast, skeleton-based methods focus on predicting hand gestures by analyzing a series of 2D or 3D key hand joint coordinates.

Compared to existing image-based methods, skeleton-based methods facilitate precise joint coordinates and support a robust hand–skeleton model based on the movements and position of the joints, which is more efficient in changing lighting conditions and occlusion. In addition, this method reduces the computational cost and enables real-time gesture interaction on mobile devices.

**Figure 1:**
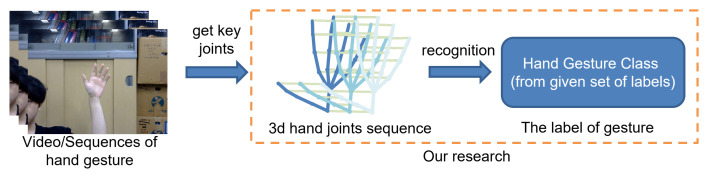
Skeleton-based hand gesture recognition.

With the rapid development of low-cost depth cameras (such as Microsoft’s Kinect) and gesture estimation (such as Openpose [8] and MediaPipe [9]), we can now easily obtain the precise coordinates of hand joint points and hand skeleton sequences. Therefore, as shown in Figure 1, in this work, a skeleton-based approach is considered.

In recent years, the graph convolutional network (GCN) has attracted widespread attention in skeleton-based dynamic hand gesture recognition due to its efficient spatio-temporal feature modeling capabilities. The spatio-temporal graph convolutional network (ST-GCN) proposed by Yan et al. [10] captures spatial correlation by utilizing bone topology while modeling the temporal relationship between joints through convolution operations in the temporal dimension. However, existing GCN-based methods still face two major challenges: first, the fixed predefined graph ignores the implicit relationship of non-adjacent joints, limiting the modeling of complex spatial features; second, temporal modeling is limited to local convolution, making it difficult to capture long-term dependencies.

A transformer has significant advantages in modeling long-range dependencies. Vision Transformer (ViT) [11] has achieved outstanding results in the field of computer vision, and its flexibility makes it a potential method to explore spatial and temporal modeling in dynamic hand gesture recognition. However, there are also shortcomings when it is directly applied to hand gesture recognition, including neglect of local spatial relationships, insufficient adaptability to bone topology, and over-fitting problems on small-scale datasets. This limits its ability to understand gesture characteristics.

To address these problems, this paper proposes a novel neural network architecture named ST-KT, a spatio-temporal graph convolution network and transformer with the Kolmogorov–Arnold Network (KAN) [12] model, which is designed for skeleton-based dynamic hand gesture recognition. The structure combines the spatio-temporal graph convolutional network (ST-GCN) [10] and the spatio-temporal transformer module to extract the spatial and temporal features of hand gestures. By building a fully connected graph of the hand, ST-GCN is used to extract the primary features of hand gestures, while the spatio-temporal transformer module with the Kolmogorov–Arnold Network (KAN) includes a spatial KAN–Transformer (S-KT) and a temporal KAN–Transformer (T-KT), which are responsible for modeling the spatial and temporal correlations between joints. Through the fully connected layer and softmax classification, the final gesture prediction result can be obtained. The network architecture was validated on three benchmarks: the SHREC’17 track [13] dataset, DHG-14/28 [7] dataset, and Leap Motion Dynamic Hand Gesture (LMDHG) dataset [14]. Finally, we developed and tested a demonstration system based on our framework. The system uses a standard monocular PC webcam to capture RGB video streams and then uses Mediapipe hands to extract the skeleton data to achieve an acceptable latency and frames per second, as well as a high accuracy.

### 1.3. Contributions

Overall, the main contributions of this paper can be summarized as follows:

1. A novel model spatio-temporal KAN–Transformer (ST-KT) is presented for skeleton-based hand gesture recognition. To achieve this, the primary features are extracted by ST-GCN and spatial and temporal features are extracted using the self-attention mechanism.
2. A spatio-temporal KAN–Transformer encoder was designed to offer stronger nonlinear fitting capabilities and reduce the parameters.
3. Extensive experiments were performed on the SHREC’17 track and DHG datasets to demonstrate that our method achieves competitive results. The experimental results showed that the proposed model ST-KT achieved state-of-the-art (SOTA) performance on the SHREC’17 track dataset with 14 labels.
4. We also introduce a dynamic hand gesture recognition demonstration system based on our framework to test the actual use-case effects of our model. It effectively shows that a standard PC webcam can fully collect and extract hand information to realize skeleton-based hand gesture recognition.

The rest of the paper is organized as follows: Section 2 outlines related work on GCN, self-attention, and transformer-based hand gesture recognition methods. Section 3 describes our proposed method and architecture. Section 4 discusses experimental results and performance evaluation. Section 5 presents a demonstration system based on our framework. Finally, we summarize the results and discuss potential future research directions in Section 6.

## 2. Related Works

In this section, we primarily focus on existing works that are closely related to ours, including dynamic hand gesture recognition based on 3D skeleton data as well as the application of Graph Convolutional Networks (GCNs), Transformers, and Kolmogorov–Arnold Networks (KANs) in hand gesture recognition. To achieve this, we reviewed various hand gesture recognition methods, analyzing their strengths, limitations, and capabilities for different applications. These methods are classified into five main categories: color gloves, skin color, appearance, depth, and skeleton-based techniques.

**Color gloves method** [15]: This method uses colored gloves and usually implements hand gesture recognition through hand-crafted features. This method is simple and low-cost, but it requires additional wearable equipment, restricts the natural movement of people, and is severely affected by environmental conditions such as background color.

**Skin color method** [16,17]: This method uses the unique color properties of the human skin to distinguish between hands within the visual frame. Although it is low-cost and simple, this method has major drawbacks due to lighting changes and individual skin color differences. Similar to the color gloves method, it is also severely affected by environmental conditions such as background color.

**Appearance-based method** [18,19]: This method directly interprets images or videos and analyzes detailed visual representations of the hand. It effectively improves accuracy for static gestures. However, dynamic gestures are more challenging because they are more diverse and require a lot of computing resources and training data.

**Depth-aware methods** [2,20,21]: This method uses depth sensors to create a 3D representation of the hand, providing excellent accuracy and adaptability to lighting and noise. However, high cost and computational requirements limit its widespread adoption.

**Skeleton-based method** [7,22]: This method models the skeletal structure of the hand and is robust to complex backgrounds, occlusions, and lighting changes. It stands out by its ability to standardize hand gesture recognition processing through preprocessing, ensuring consistent performance in different scenarios, and it can recognize gestures with a high accuracy.

### 2.1. Skeleton-Based Dynamic Hand Gesture Recognition

Hand gesture recognition based on skeletal data has been thoroughly researched, yet it is a challenging task. Conventional methods aim to encode hand skeleton sequences into descriptors that represent hand features. For instance, Smed et al. (2016) [7] primarily utilized the joint connections of hand skeletons captured by depth cameras, designed spatial combined features, encoded temporal characteristics, and subsequently employed linear support vector machine (SVM) classifiers for hand gesture classification. Kuznetsova et al. (2013) [23] used depth images to extract rotation, translation, and scale-invariant features and employed a multi-layer random forest (MLRF) to classify the features. Marin et al. (2014) [24] utilized the fingertip position and direction information provided by Leap Motion to calculate a temporary feature set and classified it using a multi-class SVM. However, these manually crafted features suffer from inaccuracies or robustness for reliable identification.

Skeleton-based hand gesture recognition has become the center of research in recent years, receiving significant attention, particularly with the recent advancements in deep learning techniques. This has led to the development of various advanced methods based on skeleton data. With the application of deep neural networks, significant performance improvements were achieved in this work. Deep learning-based methods encode hand skeleton sequences into coordinate vectors and feed them into deep neural networks such as RNNs, CNNs, and GCNs to extract spatial and temporal features for hand gesture recognition. For example, Juan C et al. (2018) [25] utilized convolutional neural networks (CNNs) and long short-term memory (LSTM) to learn spatial and temporal features from hand joint sequences for hand gesture classification. Chen et al. (2019) [26] proposed a novel network architecture that extracts the motion features of finger and global hand movements, which are then applied to bidirectional neural networks to identify hand gestures and enhance classification performance.

### 2.2. GCN in Hand Gesture Recognition

Methods based on GCN can effectively utilize a spatio-temporal context. Yan et al. (2018) [10] were the first to employ GCN on skeletal data. Their proposed spatio-temporal graph convolutional network (ST-GCN) constructs spatio-temporal graphs from 3D skeletons, allowing for the automatic learning of spatial and temporal patterns from data, thereby enhancing its expressive power and generalization capability. Zhang et al. (2020) [27] introduced a dual-stream graph attention convolutional network with spatio-temporal attention mechanisms, utilizing novel temporal graph attention modules and spatial graph attention modules to model temporal dependencies and construct dynamic skeletal graphs. AS-GCN (2019) [28] introduced new modules to the ST-GCN architecture to capture action links and structural links, extending existing skeletal graphs to represent high-order dependencies. 2S-AGCN (2019) [29] proposed a novel dual-stream framework, also employing Adaptive GCN (AGCN) (2018) [30], which can simultaneously model first-order and second-order information, thereby improving recognition accuracy.

However, methods based on GCN focus on the relationships among local joints. These methods require skeletal sequences to have predefined spatio-temporal graph structures, which can only characterize the coordinated movements of directly connected joints. For two distantly located joints in the hand, their dependencies cannot be effectively utilized. Moreover, GCN is not suitable for learning long-term relationships, which may reduce recognition accuracy.

### 2.3. Transformers in Hand Gesture Recognition

The transformer network, introduced by Vaswani et al. (2017) [31], is an outstanding neural network model primarily introduced for sequence modeling in natural language processing (NLP). Due to its self-attention mechanism, it performs better feature extraction than recurrent models and is excellent in handling very long sequences and parallel computation. In STA-GCN (2020) [27], spatial and temporal self-attention modules are utilized to learn trainable adjacency matrices. Chen et al. (2019) [22] proposed a hand gesture recognition method called dynamic graph spatio-temporal attention (DG-STA). It constructs dynamic spatio-temporal graphs by automatically learning node features and edges through self-attention mechanisms in spatial and temporal domains. Plizzari et al. (2021) [32] introduced a dual-stream network where the spatial self-attention module (SSA) comprehends intra-frame interactions among different body parts, while the temporal self-attention module (TSA) models inter-frame correlations.

The transformer network has been applied to various popular computer vision tasks. Most hand gesture recognition research works are based on RGB images or biosignals, facing greater challenges in handling complex backgrounds and various noise conditions. Our proposed skeleton-based transformer network reduces computational costs and improves real-time efficiency with better recognition performance and robustness.

### 2.4. Kolmogorov–Arnold Networks

Kolmogorov–Arnold Networks (KANs) [12], inspired by the Kolmogorov–Arnold representation theorem, is a neural network architecture. This theorem establishes that any multivariate continuous function can be expressed as a sum of univariate continuous functions, providing a robust mathematical framework for the representation and computation of complex functions.

Compared to conventional CNNs, KAN offers advantages in terms of reduced parameter amount and computational complexity, making it an excellent choice for scenarios where efficient models are required. The comparison of MLP and KAN is shown in Table 1. It stands as a promising alternative to traditional neural network models. Despite this, the application of KAN in image processing, particularly in addressing issues of high computation and memory usage, remains largely unexplored.

Our contribution builds upon KAN by introducing improvements and applying them to the domain of hand gesture recognition. By integrating KAN with the transformer architecture as KAN–Transformer, we leverage its strength in modeling nonlinear relationships. The enhancements to KAN not only reduce the number of model parameters and decrease computational complexity but also address the limitations of transformers in local information extraction. This combined approach significantly enhances the overall model performance.

## 3. Methodology

**Figure 2:**
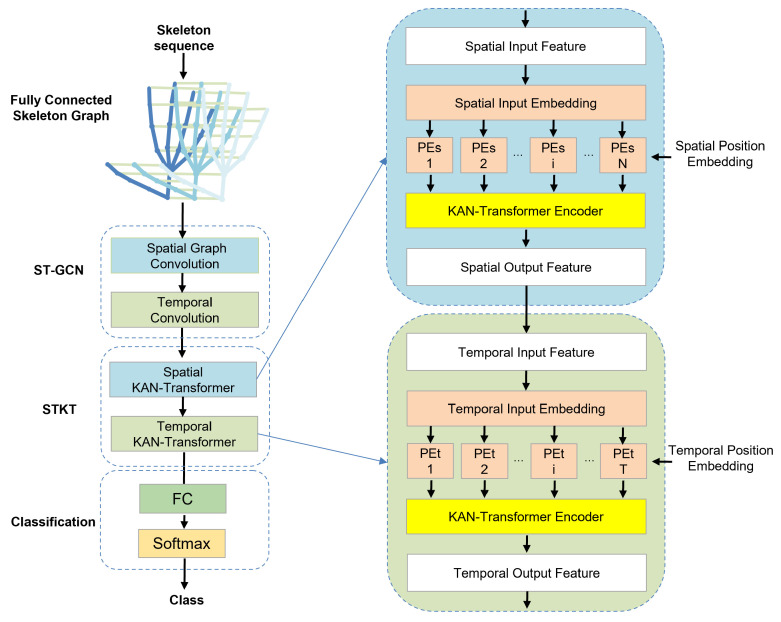
ST-KT, proposed method.

As illustrated in Figure 2, the proposed spatio-temporal graph convolution network and transformer with Kolmogorov–Arnold Network (KAN) model is composed of spatio-temporal graph convolutional network (ST-GCN) modules and spatio-temporal KAN–Transformer modules (ST-KT).

The input data of the ST-KT model include hand joint point coordinates, represented by dimensions (B,C,T,N), where *B* represents the batch size, *C* represents the joint 3D coordinates, *T* represents the number of frames of the gesture video, and *N* represents the number of hand joint points.

Firstly, to represent the gesture data as a graph structure, a fully connected skeleton graph is constructed from the input hand skeleton sequence. The ST-GCN module is used to extract the primary features from the skeleton sequence. Then, the spatial and temporal features are further extracted through the spatio-temporal transformer module. This module contains a spatial KAN–Transformer (S-KT) and a temporal KAN–Transformer (T-KT), which focus on spatial and temporal features, respectively. The number of input channels to the model is set to 128, 128, 128, 256, 512. For the transformer encoder, a critical component, the number of Multi-Head Self-Attention (MSA) heads is defined as 8, while the number of layers in each module is denoted by *L*. The configuration enables the model to better learn the relationship between nodes in the gesture sequences and more effectively capture the dynamic changes of gestures.

By integrating all the key techniques, the ST-KT model is capable of better capturing spatio-temporal information in hand gesture recognition tasks and enhances the ability to represent hand gesture sequence features, thereby achieving a more accurate classification.

### 3.1. Fully Connected Skeleton Graph Construction

**Figure 3:**
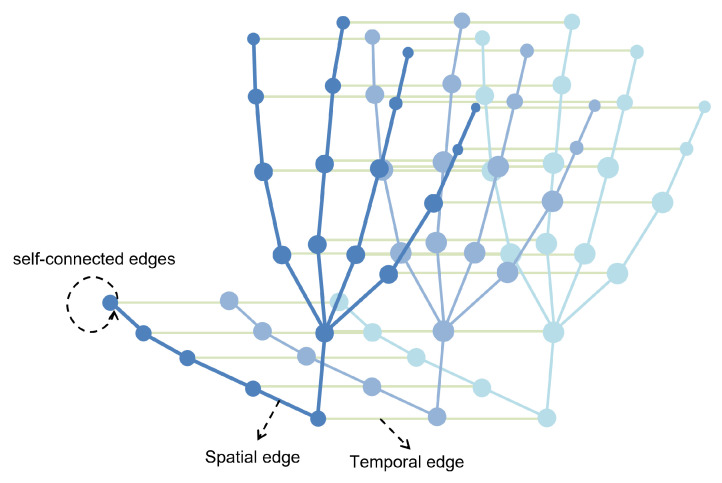
Fully connected skeleton graph. The blue connections represent spatial edges, and the green connections represent temporal edges.

A fully connected skeleton graph as shown in Figure 3 is constructed from the input hand skeleton sequence. This step serves as the basis for the model input, representing the gesture data as a graph structure, where nodes represent the key skeleton points of the hand and edges represent their connection relationships. Based on the hand skeleton graph, GCN considers the joint feature information. Joint data represent the joint point information that constitutes the hand structure, usually in the form of 3D coordinates.

The process involves extracting *N* hand joints from each frame to represent the hand skeleton in a given video comprising *T* frames. Subsequently, a fully connected skeleton graph G=(V,E) is constructed from this sequence of hand skeletons. Here, V={v(t,i)|t=1,…,T,i=1,…N} denotes the set of nodes, where v(t,i) represents the *i*th hand joint at time step t. Node features are represented by F={f(t,i)|t=1,…,T,i=1,…N}, with f(t,i) indicating the feature vector of node v(t,i), extracted from the 3D coordinates of the nodes. It is noteworthy that each node is connected to all other nodes, including itself. For clarity, we define three types of edges on the edge set *E* as follows.

The step of connecting two different nodes at the same time represents spatial edges.

(1) v(t,i)→v(t,j)(i≠j)

Connecting two nodes at different time steps represents temporal edges.

(2) v(t,i)→v(k,j)(t≠k)

Connecting nodes to themselves represents self-connected edges.

(3) v(t,i)→v(t,i)

Through the construction of the spatio-temporal graph, the hand skeleton sequence can be represented as a hierarchical structure containing spatial and temporal connections between joints. This structure can adequately represent the spatio-temporal relationship between joint points in the hand skeleton sequence, enable each joint node to collect the local information of adjacent joint nodes, and capture the changes in joint sequences over time. By utilizing the structure of the spatio-temporal graph, the ST-GCN module can extract primary features of the hand skeleton graph. These primary features include the spatial position information of the joints, the motion trajectory information, and the spatial relationship information between adjacent joints.

### 3.2. Spatio-Temporal Graph Convolutional Network Module

The structure of the hand skeleton graph contains rich feature information. Each joint point can gather local information from nearby joints by connecting different bones, thereby directly obtaining more useful information. Therefore, in the ST-KT model, the ST-GCN module is designed to extract the primary features of the hand skeleton graph.

The structure of ST-GCN is a hierarchical stack of spatio-temporal blocks consisting of spatial graph convolution (S-GCN) layers and temporal convolution (TCN) layers. ST-GCN was proposed by Yan et al. [10].

The S-GCN operation aggregates feature vectors of adjacent joints in the spatial domain, followed by weight-sharing convolutional operations. For the input *X*, the S-GCN operation can be expressed as follows:

(4) $$\hat{A}=D^{-\frac{1}{2}}*\left(A+I\right)*D^{-\frac{1}{2}}$$

(5) $$X_{s}=\hat{A}*X*W$$

*A*: adjacency matrix, *D*: degree matrix of *A*, *W*: trainable weight matrix.

Unlike spatial graph convolution, the temporal relationships are linear; hence, ordinary convolution operations are typically used instead of graph convolutions to capture temporal relations.

(6) $$X_{st}=\mathrm{Conv}2D\left(X_{s},\;kernel_{size}=k,\;padding=\prime same\prime \right)$$

### 3.3. Transformer Encoder with KAN

**Figure 4:**
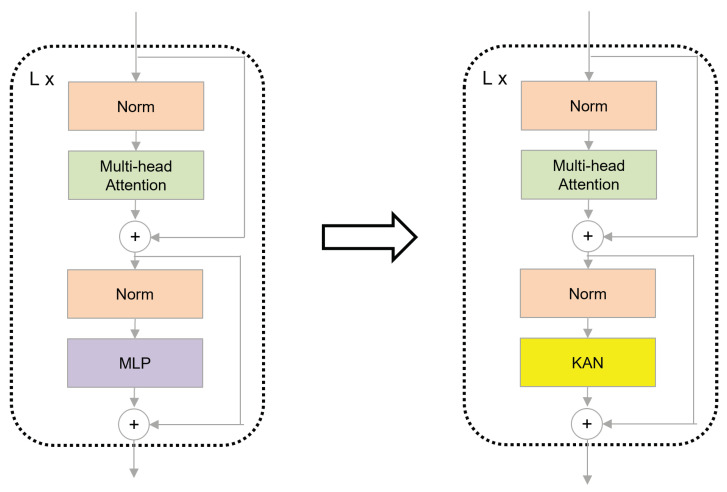
Vision transformer encoder (ViT) [11] (**left**) and the proposed KAN–Transformer encoder block (**right**).

The KAN–Transformer encoder shown as Figure 4 consists of a multi-head self-attention (MSA) block, Kolmogorov–Arnold Network (KAN) block, and layer normalization (LN). Residual connections are applied to prevent gradient-related performance issues.

For each head of MSA, the self-attention mechanism plays an important role in modeling the relationship between sequence elements. From the input sequence $X\in R^{N\times C}$, $Q,K,V\in R^{N\times d}$ are calculated as follows:

(7) $$Q,K,V=XW_{Q},XW_{K},XW_{V}$$

where $W_{Q},W_{K},W_{V}\in R^{C\times d}$ are learnable weight matrices and *d* is the dimension of *K*. By calculating the dot product of *Q* and *K* and then properly normalizing by the scaling factor $\frac{1}{\sqrt{d}}$, the output of attention can be expressed as follows:

(8) $$\mathrm{Attention}\left(Q,K,V\right)=\mathrm{Softmax}\left(\frac{QK^{T}}{\sqrt{d}}\right)V$$

Then, each head performs a self-attention operation in parallel, using different learnable parameters to obtain Q,K,V. The MSA output is the concatenation of the outputs of all attention heads, which is represented as follows:

(9) $$\mathrm{MSA}\left(Q,K,V\right)=\mathrm{Concat}\left(Y_{1},Y_{2},\ldots ,Y_{h}\right)W_{out}$$

where *h* is the number of attention heads, and $Y_{i}$ represents the output of the *i*th self-attention, i∈{1,2,…,h}. $W_{out}$ is a trainable matrix.

In our modal, the original node features *F* extracted from the coordinates of the hand skeleton lack spatial identity and temporal information, which describe the corresponding hand joint and the time step of each node, respectively. To address this deficiency, we propose the spatio-temporal position embedding technique. The spatial KAN–Transformer (S-KT) and the temporal KAN–Transformer (T-KT) focus on spatial and temporal information, respectively.

(1) Spatial KAN–Transformer (S-KT): Extract key spatial features through input embedding, spatial position embedding, multiple transformer encoder layers, and global maximum pooling operations. For the input feature $X_{s}=\left\{x_{i}^{s}\in R^{1\times C}|i=1,2,\ldots ,N\right\}$, the input embedding is a trainable linear matrix $E\in R^{C\times C_{s}}$, where *C* and $C_{s}$ are input and output channels, mapped to a higher-dimensional feature vector.

(10) $$Y^{\prime }=X_{s}E$$

Then, add the output vector to the spatial position embedding $PE_{s}\in R^{N\times C_{s}}$, which is a trainable matrix and each vector corresponds to a hand joint, to obtain the spatial token embedding sequence.

(11) $$Y_{s}=Y^{\prime }+PE_{s}=X_{s}E+PE_{s}$$

The spatial transformer encoder layer includes a multi-head self-attention (MSA) module and a Kolmogorov–Arnold Network (KAN) module. The Spatial KAN–Transformer can be represented as the following operations:

(12) $${\left(y_{l}^{s}\right)}^{\prime }=\mathrm{MSA}\left(\mathrm{LN}\left(y_{l-1}^{s}\right)\right)+y_{l-1}^{s}$$

(13) $$y_{l}^{s}=\mathrm{KAN}\left(\mathrm{LN}\left({\left(y_{l}^{s}\right)}^{\prime }\right)\right)+{\left(y_{l}^{s}\right)}^{\prime }$$

where $y_{l}^{s}$ denotes the output feature sequence at the *l* layer; the global max pooling operation can better extract salient spatial features.

(2) Temporal KAN–Transformer (T-KT): Extract key temporal features through input embedding, temporal position embedding, multiple transformer encoder layers, and global average pooling operations. Similarly, for the input feature $X_{t}=\left\{x_{i}^{t}\in R^{1\times C}|i=1,2,\ldots ,T\right\}$, input embedding $E\in R^{C\times C_{t}}$ is a trainable linear matrix, the time position embedding $PE_{t}\in R^{T\times C}$ is a trainable matrix, and each vector corresponds to a joint at a certain time step.

(14) $$Y_{t}=Y^{\prime }+PE_{t}=X_{t}E+PE_{t}$$

The temporal transformer encoder layer also includes a multi-head self-attention (MSA) module and a Kolmogorov–Arnold Network (KAN) module. The temporal KAN–Transformer can be represened as the following operations:

(15) $${\left(y_{l}^{t}\right)}^{\prime }=\mathrm{MSA}\left(\mathrm{LN}\left(y_{l-1}^{t}\right)\right)+y_{l-1}^{t}$$

(16) $$y_{l}^{t}=\mathrm{KAN}\left(\mathrm{LN}\left({\left(y_{l}^{t}\right)}^{\prime }\right)\right)+{\left(y_{l}^{t}\right)}^{\prime }$$

where $x_{l}^{t}$ denotes the output feature sequence at the *l* layer. The global average pooling operation can preserve smooth features between time steps and is used to handle long- and short-term time dependencies.

### 3.4. Classification

We integrate the Spatial KAN–Transformer Stream (S-KT) and the Temporal KAN–Transformer Stream (T-KT) to form the spatio-temporal transformer network (ST-KT). These architectures are designed to comprehensively capture the spatial and temporal correlations within hand gesture sequences, thereby enhancing the overall performance of skeleton-based hand gesture recognition systems.

ST-KT combines the features of S-KT and ST-GCN. The calculation formula is as follows:

(17) $${\mathrm{ST}}_{\mathrm{out}}=\mathrm{MLP}\left(\mathrm{TKT}\left(\mathrm{SKT}\left(Y_{\mathrm{st}}\right)\right)\right)$$

Classification using standard *cross-entropy* loss and use of the *Softmax* classifier.

(18) $$\mathrm{Pred}=\mathrm{softmax}\left({\mathrm{ST}}_{\mathrm{out}}\right)$$

## 4. Experiments and Results

In Section 4.1, we provide a detailed description of the datasets and training protocols utilized in our experiments. Following that, in Section 4.2, we discuss our training details and the conducted ablation studies to evaluate the effectiveness of each component proposed in our method. Finally, in Section 4.3, we report the experimental results and compare them with relevant approaches.

### 4.1. Datasets

#### 4.1.1. SHREC’17 Track

SHREC’17 track [13] is a benchmark dataset for hand gesture recognition that includes two types of hand gesture configurations. The first configuration involves 14 hand gestures that are captured using an Intel RealSense depth camera, focusing on finger articulations. The second configuration consists of 28 hand gestures that are executed with whole hand movements. Each hand gesture is performed by 28 participants, with each participant repeating each hand gesture from 1 to 10 times, resulting in a dataset of 2800 sequences. For our experiments, the dataset comes with a predefined split of the data into 70% for training and 30% for testing.

The hand gestures included in the dataset cover a range of manipulations, such as grab (G), expand (E), pinch (P), rotation clockwise (RCW), rotation counterclockwise (RCCW), tap (T), swipe right (SR), swipe left (SL), swipe up (SU), swipe down (SD), swipe X (SX), swipe plus (S+), swipe V (SV), and shake (Sh). To cater to the needs of human–computer interface applications, we classify these 14 hand gestures into fine-grained and coarse-grained classes. Fine-grained hand gestures represent changes in hand shape, while coarse-grained hand gestures pertain to the overall movement of the hand. Each sequence in the dataset comprises depth images with a resolution of 640 × 480 pixels, along with the 3D world space and 2D image space coordinates of 22 hand joint positions.

#### 4.1.2. DHG Dataset

The DHG dataset [7] is a hand gesture recognition dataset that contains sequences of multiple hand gestures, and it is typically used together with SHREC’17 track since they have the same categories of gestures but different data splitting. The main similarities and differences with SHREC’17 track are shown in Table 2. In this dataset, each hand gesture is performed by multiple participants using different fingers or the entire hand, increasing the diversity and challenge of the data. The data collection was performed using Intel RealSense cameras, providing 3D coordinate information for each hand gesture on 22 joint points.

When evaluating models, the DHG dataset typically uses leave-one-out cross-validation, where for each hand gesture, the data from 19 participants are used for training, and the remaining 1 participant’s data are used for testing. This approach yields the average recognition accuracy for each hand gesture, and then the accuracy of all hand gestures is averaged to obtain the final model evaluation result.

This dataset is highly valuable for hand gesture recognition research due to its rich hand gesture data and the diversity of the data increased by different performance methods, aiding models in generalization under a wider range of conditions. It is also one of the benchmark datasets for many hand gesture recognition research works.

#### 4.1.3. Leap Motion Dynamic Hand Gesture (LMDHG) Dataset

The Leap Motion Dynamic Hand Gesture (LMDHG) dataset [14] is a publicly available dataset designed for dynamic hand gesture recognition tasks. It was created using the Leap Motion controller, which accurately tracks hand and finger movements in 3D space. This dataset contains 608 gesture sequences of 13 dynamic gestures performed by 25 subjects, each containing a total of 46 hand joints for both hands (23 joints for each hand). Among them, 415 sequences are used for training, and 194 sequences are used for testing.

### 4.2. Training Details

Our proposed network was implemented based on the PyTorch platform. We utilized the SDG optimizer with a Nesterov momentum of 0.9. The initial learning rate used to train our model was $1\times 10^{-3}$. During model training, we employed the Cosine-Annealing-Warm-Restarts learning rate schedule. The learning rate gradually decreased from a higher value to a lower value in a cosine curve and then returned to the initial learning rate in a new cycle. The initial restart occurred at 10 epochs, with subsequent restart intervals doubled. The minimum learning rate was set to $1\times 10^{-6}$, ensuring smooth convergence and effective learning rate adjustments throughout training. And we set the batch size to 32 with a dropout rate of 0.2. The number of encoders in our model was set to 6, with 8 heads for multi-head attention. To enhance training stability and generalization performance, we set the minimum sequence length to 180 for the SHREC’17 track and DHG datasets, and to 250 for the LMDHG dataset. We set the number of hand joints to 22 for the SHREC’17 track and DHG datasets and 46 for the LMDHG dataset.

Additionally, to enhance the robustness and generalization of our skeleton-based hand gesture recognition model, we implemented a comprehensive data augmentation strategy. This strategy is designed to simulate a wide range of realistic scenarios that the model might encounter in practical applications. The augmentation techniques employed are as follows:

1. **Scaling**: Scale the skeleton data to represent different body types. This variation addresses the diversity of human body types and ensures the model is valid across a wide range of people. We uniformly scale the coordinates of the skeleton joints by a factor randomly selected from a predefined range, simulating changes in the user’s physical size.
2. **Shift**: Shift the skeleton data horizontally and vertically to represent different initial positions relative to the sensor. This shift is performed by adding a constant offset (randomly selected from a set range) to the joint coordinates to ensure that the model remains valid regardless of the user’s position within the sensor’s field of view.
3. **Noise injection**: Real-world measurements often contain noise due to sensor inaccuracies or slight involuntary movements. We simulated these conditions by adding Gaussian noise to the skeleton joint coordinates. The noise parameters (mean and standard deviation) were chosen based on empirical observations of sensor noise characteristics in preliminary testing.
4. **Temporal interpolation**: Temporal interpolation was applied to the skeleton sequence to simulate different speeds of gesture execution. This method involves subsampling or oversampling the time frames in the sequence, speeding up or slowing down the motion. In addition, we set all input time lengths T to 180.
5. **Rotation**: Given that users may interact with the sensor from various directions, our model must be able to accurately interpret gestures from multiple angles. We achieved this by rotating the skeleton data around the vertical axis. The rotation angle is randomly selected from a specified range, allowing the model to learn and predict gestures that are not affected by changes in orientation.

During the training phase, each augmentation technique is applied individually or in combination to create a robust dataset that simulates a variety of real-world conditions. The augmented dataset significantly improves the performance of the model, especially in its ability to generalize across different users and environment settings.

### 4.3. Ablation Study

**Figure 5:**
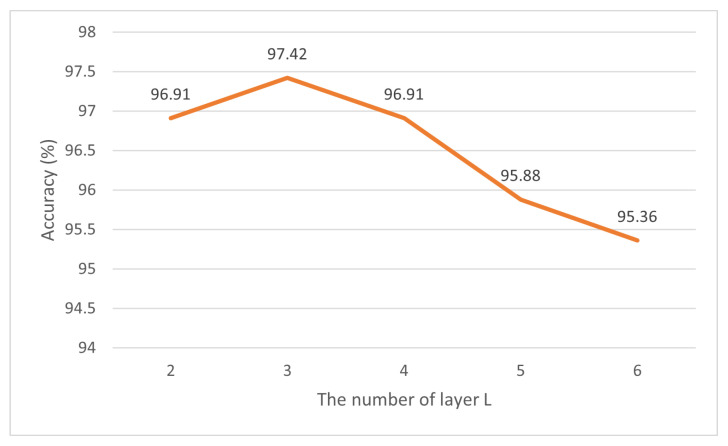
Comparison of different encoder layers on the LMDHG dataset.

We investigated how different operations on model architecture and data affect the performance of our method. Our approach consists of three main components, including the spatio–temporal graph convolutional network module (ST-GCN); the spatio–temporal transformer (STTR, STR + TTR), which consists of the spatial transformer (STR); and the temporal transformer (TTR), position encoding, and the Kolmogorov–Arnold Network. In this section, we validate the effectiveness of these components. All experiments were conducted on the SHREC’17 track dataset and are shown in Table 3 and Table 4. The number of transformer encoder layers was also compared on the LMDHG dataset, and the results are shown in Figure 5.

#### 4.3.1. Effectiveness of Basic Module

We first examined the performance of all module combinations, as shown in method D, and then we gradually removed single modules to examine their effects, as shown in methods A, B, and C. As shown in Table 3, deleting the ST-GCN modules causes the gesture accuracy of the 14-label to drop by 0.7%, which indicates that the feature representation is enhanced by extracting primary features through the ST-GCN module. The extraction of primary features is essential for enhancing the capabilities of sequence modeling and feature extraction in hand gesture recognition. These features typically encompass skeletal coordinates, joint angles, velocity, and acceleration, providing a foundational understanding of the dynamic nature of hand gestures. The precise extraction of these elements facilitates the capture of nuanced hand gesture variations, thereby enhancing the recognition accuracy and robustness.

#### 4.3.2. Modification of Transformer Encoder

The effectiveness of spatio-temporal position encoding was verified. We experimented with four PE variants: no position encoding, spatial position encoding, temporal position encoding, and spatio-temporal position encoding. As shown in Table 4, the spatio-temporal position encoding version achieved the highest recognition accuracy. This further proves the importance of spatial- and temporal-order data for each joint.

The experimental results also verify the effectiveness of different neural network architectures in the transformer encoder. As shown in Table 4, KAN achieves a higher recognition accuracy than MLP. This result shows that KAN, as an emerging architecture, has advantages in specific task performance and provides new possibilities for the future development of transformers.

#### 4.3.3. Performance of Different Layer Numbers

In addition, we also compared the performance of transformer encoders with different layers using the LMDHG dataset. According to the results shown in Figure 5, when the number of encoder layers increases to 3, the recognition accuracy reaches the highest accuracy of 97.42%. However, further increasing the number of layers will lead to overfitting of the training data and poor performance on the test data. In order to avoid the over-smoothing problem, three layers were selected as the optimal number of encoder layers for further analysis.

### 4.4. Comparison with Relevant Approaches

We compared our method with relevant approaches on the SHREC’17 track dataset, DHG dataset, and LMDHG dataset. The SOTA methods include methods utilizing GCN and methods employing self-attention and a transformer. The GCN-based methods include spatio-temporal graph convolutional network methods ST-GCN (2018) [10] and spatio-temporal dynamic attention graph convolutional network STDA-GCN (2024) [33]. The transformer-based methods include spatio-temporal attention network methods based on dynamic graphs DG-STA (2019) [22], decoupled spatio-temporal attention network DSTA-Net (2020) [34], the method with multi-scale multi-head attention module proposed by Li et al. (2024) [35], and spatial graph convolutional and temporal attention-based methods STr-GCN (2023) [36]. The results are shown in Table 5, Table 6 and Table 7.

Our model achieved an excellent accuracy of 97.5% on the 14-label dataset, outperforming the SOTA methods, and 94.1% on the 28-label dataset. This demonstrates the ability of our model to capture and analyze complex relationships and dynamic changes in skeleton sequences. In particular, the high accuracy on the 14-label dataset highlights the robustness of our model and its adaptability to various gesture complexities.

The 14-label classification task is a relatively coarse-grained classification task, and the differences between categories are more significant. The 28-label classification task is a fine-grained classification, involving subtle differences in the use of fingers in the same gesture action. This classification relies more on the identification of local details.

Our model achieved the best performance in the 14-label gesture recognition task, indicating strong overall feature extraction and modeling capabilities. Its global modeling capabilities enable it to capture the overall dynamic features of gestures well, so it performs well in this coarse-grained task. However, it may be because the feature extraction of our current model is mainly focused on the overall spatio-temporal pattern and lacks specific optimization for subtle finger movements, and it is less sensitive to local features. It performs worse than the GCN-based method on 28-label.

**Figure 6:**
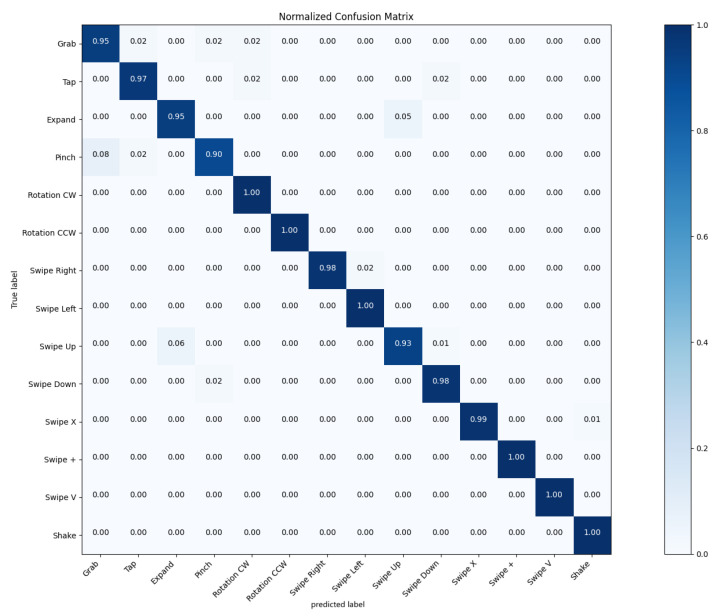
The confusion matrix of the SHREC’17 track dataset when using 14 hand gesture classes.

We also examined the performance of a multi-class hand gesture recognition model through the analysis of its confusion matrix. As shown in Figure 6, the model demonstrates high overall accuracy on 14 labels, with particularly strong performance in certain classes while showing some limitations in others. The confusion matrix reveals a high level of accuracy across most classes, as evidenced by the predominantly dark blue diagonal elements. This indicates that the model is generally successful in correctly classifying the various hand gestures. The off-diagonal elements of the matrix show very low values, indicating minimal confusion between different classes. This suggests that the model successfully learned to differentiate between the various hand gestures with high precision.

Several classes showed near-perfect recognition rates, and the majority of classes demonstrated strong performance with accuracy rates above 0.90. While still showing good performance, the Pinch class had a slightly lower accuracy (0.90) compared to other hand gestures. This may indicate that Pinch gestures share some features similar to those of other classes, making them marginally more challenging to distinguish. In addition, by analyzing the gesture sequences of the dataset, we believe that there are two main reasons: (a) measurement errors caused by inaccurate skeletal joint collection and (b) limitation of sample number.

**Figure 7:**
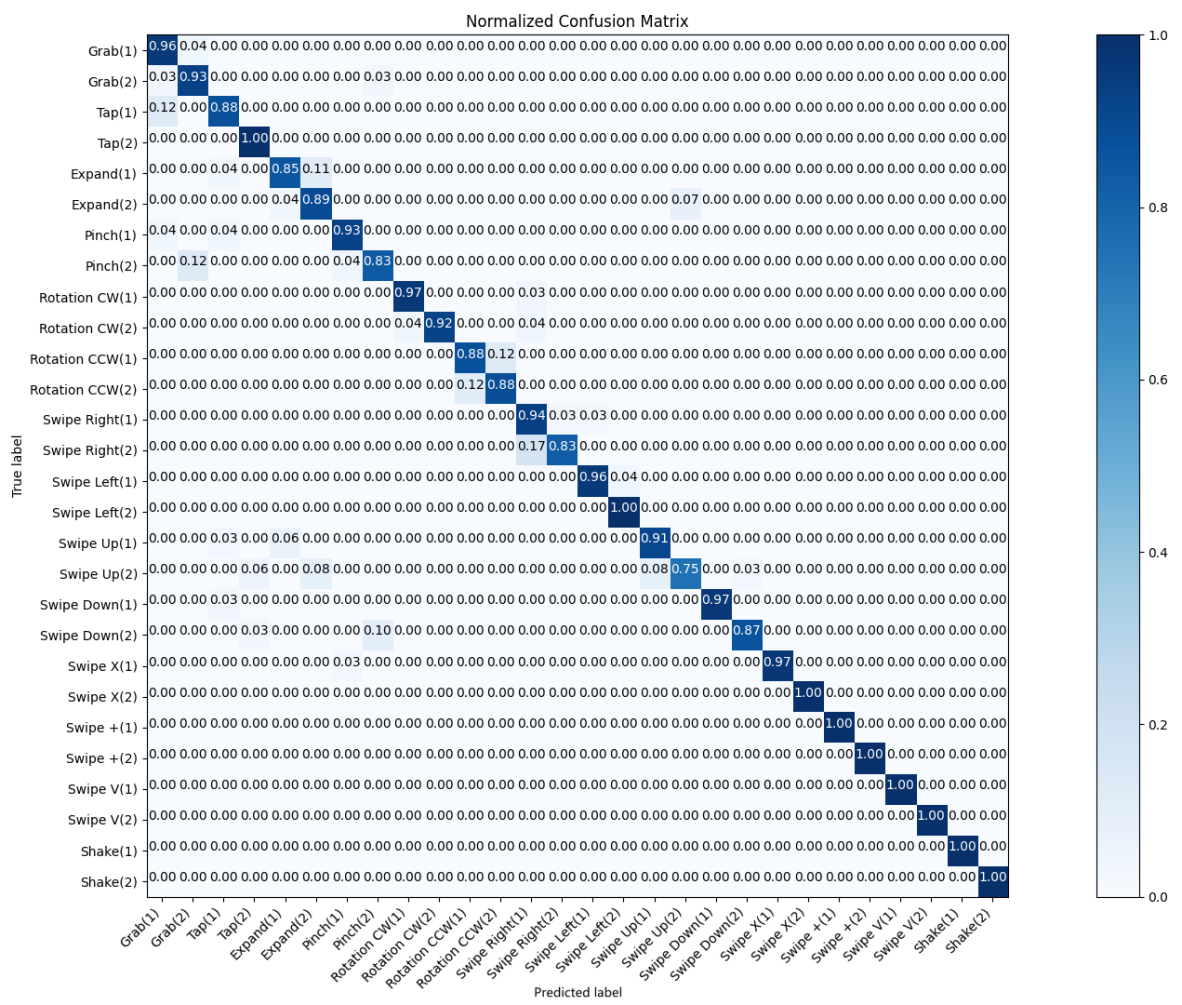
The confusion matrix of the SHREC’17 track dataset when using 28 hand gesture classes.

As shown in Figure 7, the confusion matrix for the hand gesture recognition model with 28 labels reveals both its strengths and areas needing improvement. The matrix’s diagonal, representing correctly classified gestures, indicates a high overall accuracy, suggesting the model’s effectiveness in recognizing diverse gestures.

Our model faced challenges in differentiating between certain gesture pairs, such as “Grab” and “pinch”, and “swipe up” and “expand”. These confusions may arise from the visual or dynamic similarities between these gestures.

When dealing with inter-category confusion, our model exhibited confusion within specific categories, notably “Rotation CCW” and “swipe left”. This indicates the need for more refined feature extraction to capture subtle differences within gesture categories.

Our proposed model achieved an accuracy of 94.3% on the DHG-14 dataset, surpassing the best performance of the STDA-GCN model, and achieved 91.0% on DHG-28. This demonstrates that our model is able to capture complex spatio-temporal patterns and interactions in skeletal sequences more effectively. The superior performance, particularly on the DHG-14 dataset, underscores the robustness and adaptability of our model to variations in the dataset.

The results in the table highlight the effectiveness of our model in handling complex hand gesture recognition tasks. Despite the slower training speed, the model’s performance indicates strong generalization capabilities, especially when faced with diverse and new data. This trade-off between model size and accuracy suggests that our approach is well-suited for applications where a high recognition accuracy is paramount, even if computational resources are less constrained.

We compare our method with related methods on the LMDHG dataset. The state-of-the-art methods include the framework MMEGRN (2023) [39], which combines multiple sub-networks (ConvLSTM, TCN, and 3DCNN); the spatio-temporal deep convolutional LSTM model DConvLSTM (2021) [40]; and the method based on 3D pattern assembled trajectories proposed by Boulahia et al. (2017) [14]. The results are shown in Table 7.

**Table 7:** Recognition accuracy comparison with state-of-the-art methods on LMDHG dataset. The best results are shown in bold.

Model	Acc on LMDHG (%)
Boulahia et al. (2017) [14]	84.78
DConvLSTM (2021) [40]	93.81
MMEGRN (2023) [39]	95.88
Ours	**97.42**

Our proposed model achieved the highest accuracy of 97.42% on the LMDHG dataset, which is 1.54% higher than the MMEGRN method. MMEGRN combines ConvLSTM, TCN, and 3DCNN with classifiers to improve the accuracy of skeleton hand gesture recognition. By combining various feature extraction and classification techniques, MMEGRN effectively captures and classifies dynamic changes in skeletal sequences, achieving superior performance compared to previous methods. However, for this dataset with significant differences between categories, our proposed method leverages its powerful overall feature extraction and modeling capabilities to achieve a more comprehensive feature representation.

### 4.5. Discussion of Result

#### 4.5.1. Performance

We can see that the proposed ST-KT is superior to the other methods and achieves the SOTA performance under 14-label hand gesture recognition tasks on both the SHREC’17 track and DHG datasets, and also on the LMDHG dataset. The superior performance on tasks with significant differences between categories indicates that its overall feature extraction and modeling capabilities are strong, and its global modeling capabilities enable it to capture the overall dynamic characteristics of gestures well.

Compared to GCN-based methods, our model demonstrates superior performance across most coarse-grained classification tasks. This result underscores the importance of fully capturing global information. Incorporating both local and global interactions enables it to capture the overall dynamic features of gestures, resulting in an enhanced recognition accuracy.

When compared to previous transformer-based methods, our model shows improvements across all indicators. Our improvements enhance the ability to fuse local and global information, leading to significant gains in coarse-grained classification (14 labels) and slight improvements in fine-grained classification (28 labels). This suggests better generalization and a capacity to learn individual differences or subtle variations between subjects.

#### 4.5.2. Limitations

Although our proposed model achieves state-of-the-art performance on all benchmarks for 14 labels, an analysis of inter-category confusion in the 28-label confusion matrix reveals limitations in modeling spatial features. This may be because the feature extraction of our current model mainly focuses on the overall spatio-temporal pattern, lacks targeted optimization for subtle finger movements, and has insufficient recognition ability for local details. The performance on 28 labels still has a certain gap with the current state-of-the-art methods. Specifically, the modifications made to the Graph Convolutional Network (GCN) are not as effective as anticipated. This suggests that there is room for improvement in how spatial relationships and complex gesture structures are processed by the model.

The limitations in spatial feature modeling impact the model’s accuracy in distinguishing between certain gesture categories. The confusion between gestures with similar spatial arrangements highlights an area where the model struggles. This finding underscores the need for further research to enhance the model’s ability to accurately capture and interpret spatial features in skeletal data.

## 5. Experimental Demonstration

To demonstrate the performance and applicability of the proposed hand gesture recognition model, we developed and tested a demonstration system. The demonstration system is able to recognize gestures based on skeleton data extracted from a single-gesture video. Below, we provide detailed information about the system pipeline, implementation, and output interface.

### 5.1. System Overview

**Figure 8:**
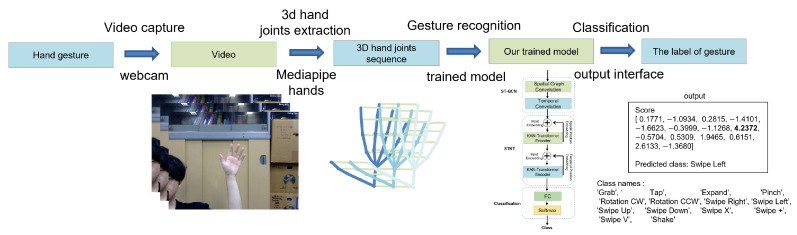
Overall structure of the demonstration system.

**Figure 9:**
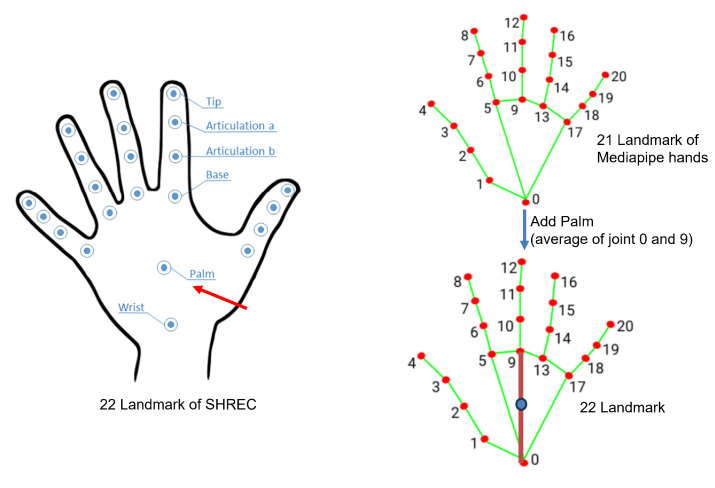
Hand skeleton extraction by Mediapipe hands.

Figure 8 illustrates the overall flow of the demonstration system. Among them, the demonstration system integrates three key components:

1. **Hand skeleton extraction module:** This module uses the Mediapipe framework to extract 3D hand skeleton data from the video stream. Each hand skeleton is represented as a sequence of 21 joints, each of which is described by its 3D coordinates. It is worth noting that since the pre-trained model uses 22 joint points after Mediapipe extracts the 3D hand skeleton points, we add a palm point by the average of the base of the middle finger and the wrist as shown in Figure 9.
2. **Hand gesture recognition model:** The ST-KT-based pre-trained hand gesture recognition model processes the skeleton sequence to classify gestures.
3. **Output interface:** A concise and easy-to-understand interface was developed to display the recognized gestures and the corresponding confidence scores.

### 5.2. Real-Time Performance

The demonstration system was tested on a laptop equipped with an AMD Ryzen 7 5800H processor and a GeForce RTX 3080 Laptop GPU.

**Figure 10:**
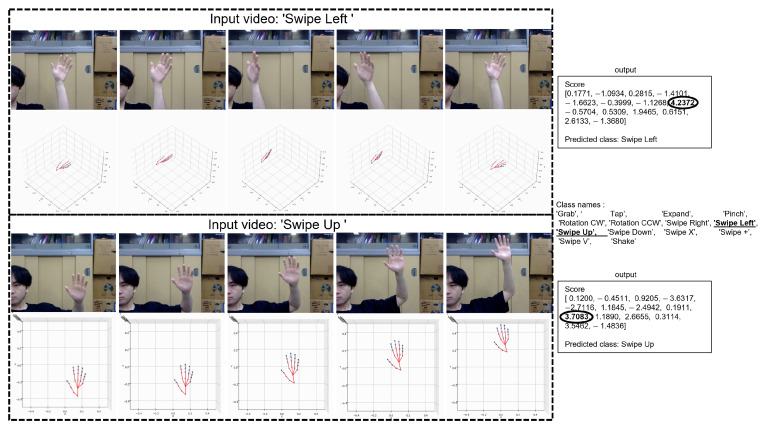
Example of the demonstration system, the circle and underline represent the highest score and the corresponding label respectively.

Observing the performance of the demonstration system, the average processing delay per frame was about 2.81 s, and the inference time was 0.37 s, which ensured a more flexible user experience. Depending on the gesture category tested, the inference classification accuracy was 75% to 99%. Some examples of the demonstration are shown in Figure 10. The system achieved a high recognition accuracy consistent with the offline evaluation results of the model.

## 6. Conclusions

### 6.1. Summary

This study proposes the ST-KT method, a novel deep learning architecture for skeleton-based hand gesture recognition. This network is a simple but effective framework that captures spatio-temporal correlations based on the spatio-temporal position encoding of the transformer and improves the nonlinear modeling capability by introducing KAN in the transformer. Our method achieved an accuracy of 97.5% on the SHREC’17 track dataset and 94.3% on the DHG dataset with 14 labels, surpassing the SOTA method. The effectiveness of the framework is demonstrated through experiments, showing its strong robustness in dealing with multiple datasets with different features and contexts.

In addition, by creating a demonstration system, we successfully demonstrated the accuracy of our framework and the fast response of the system. Since the system can run effectively on any typical personal computer with a monocular webcam, it provides a foundation for subsequent natural and inexpensive human–computer interaction.

Our findings contribute to the ongoing discussion in the field of hand gesture recognition, highlighting the potential trade-off between accuracy and computational efficiency. Future work can explore the scalability of our method in more restricted environments and larger datasets to further verify its effectiveness and efficiency.

### 6.2. Future Work

In future research, we will explore various data preprocessing techniques for 3D skeleton sequences to further improve performance. We plan to apply this method to more complex continuous hand gesture recognition tasks to study its applicability in a wider range of fields. We consider introducing an attention mechanism in the GCN component to better capture the dependencies between long-distance links in spatio-temporal graphs and further improve performance.

In addition, we plan to use continuous gesture videos as input for the demonstration system to achieve gesture segmentation and recognition. For the recognized gestures, we will consider converting them into instructions for some systems, such as computers, through a functional mapping to achieve natural and convenient human–computer interaction.

## Figures and Tables

**Table 1:** Comparison of MLP and KAN.

Model	Multi-Layer Perceptron (MLP)	Kolmogorov–Arnold Network (KAN)
Theorem	Universal Approximation Theorem	Kolmogorov–Arnold Representation Theorem
Formula	$\mathrm{MLP}\left(x\right)=\left(W_{2}\cdot \sigma_{1}\cdot W_{1}\right)\left(x\right)$	$\mathrm{KAN}\left(x\right)=\left(\Phi_{2}\cdot \Phi_{1}\right)\left(x\right)$
Structural	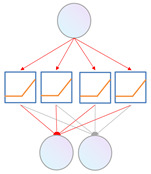	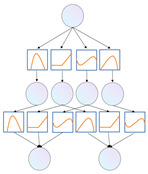
edges	learnable weights	learnable activation functions
nodes	fixed activation functions	sum operation

**Table 2:** Comparison of SHREC’17 track and DHG-14/28 datasets.

Feature	SHREC’17 track Dataset	DHG Dataset
Data Amount	2800 hand gesture sequences	2800 hand gesture sequences
Categories	14 hand gestures (single finger or entire hand)	14 hand gestures (single finger or entire hand)
Participants	28	20
Performed time of each hand gesture	between 1 and 10 times	5 times by each participants
Capture Device	Intel RealSense camera	Intel RealSense camera
Number of Joints	22 (3D coordinates)	22 (3D coordinates)
Data Splitting	70:30	Leave-one-subject-out cross-validation
Difficulty level	Stability with repetitive actions	Diversity of individual differences
Meaning	Examine effectiveness of feature extraction	Examine ability to deal with new data

**Table 3:** Ablation study for the module on the SHREC’17 track 14 label. Method D (ST-GCN + STR + TTR) achieves the highest recognition accuracy.

		STTR		
Method	ST-GCN	STR	TTR	Accuracy (%)
A	✓		✓	94.2
B	✓	✓		94.6
C		✓	✓	95.4
D	✓	✓	✓	**96.1**

**Table 4:** Ablation study for the position encoding and network structure on the SHREC’17 track 14 label. Spatio-Temporal PE and KAN achieve the highest recognition accuracy.

Class	Method	Accuracy (%)
Position Encoding	None	93.8
	Spatial PE	96.7
	Temporal PE	95.7
	Spatio-Temporal PE	**97.1**
Network Structure	MLP	96.1
	KAN	**97.5**

**Table 5:** Recognition accuracy comparison with state-of-the-art methods on SHREC’17 track dataset. The best results are shown in bold.

Method	Model	Acc on SHREC2017’ 14 Label (%)	Acc on SHREC2017’ 28 Label (%)
GCN	ST-GCN (2018) [10]	92.7	87.7
	MS-ISTGCN(2022) [37]	96.7	94.9
	TD-GCN(2023) [38]	97.0	95.4
	STDA-GCN(2024) [33]	97.1	**95.8**
Transformer	STr-GCN (2023) [36]	93.39	89.2
	DG-STA (2019) [22]	94.4	90.7
	Li et al. (2024) [35]	96.9	94.2
	DSTA-Net(2020) [34]	97.0	93.9
	Ours	**97.5**	94.1

**Table 6:** Recognition accuracy comparison with state-of-the-art methods on DHG dataset. The best results are shown in bold.

Method	Model	Acc on DHG 14 Label (%)	Acc on DHG 28 Label (%)
GCN	ST-GCN (2018) [10]	91.2	87.1
	MS-ISTGCN (2022) [37]	93.7	91.2
	TD-GCN (2023) [38]	93.9	91.4
	STDA-GCN (2024) [33]	94.2	**92.1**
Transformer	DG-STA (2019) [22]	91.9	88.0
	DSTA-Net (2020) [34]	93.8	90.9
	Li et al. (2024) [35]	94.2	**92.1**
	Ours	**94.3**	91.0

## Data Availability

The data provided in this study are available upon request by contacting the corresponding author.

## References

[1] Kim M., Cho J., Lee S., Jung Y. (2019). IMU sensor-based hand gesture recognition for human-machine interfaces. Sensors.

[2] Linqin C., Shuangjie C., Min X., Jimin Y., Jianrong Z. (2017). Dynamic hand gesture recognition using RGB-D data for natural human-computer interaction. J. Intell. Fuzzy Syst..

[3] Rautaray S.S., Agrawal A. Interaction with virtual game through hand gesture recognition. Proceedings of the 2011 International Conference on Multimedia, Signal Processing and Communication Technologies.

[4] Rautaray S.S., Agrawal A. (2015). Vision based hand gesture recognition for human computer interaction: A survey. Artif. Intell. Rev..

[5] Deafness and Hearing Loss. https://www.who.int/news-room/fact-sheets/detail/deafness-and-hearing-loss.

[6] Murad B.K., Alasadi A.H.H. (2024). Advancements and Challenges in Hand Gesture Recognition: A Comprehensive Review. J. Electr. Electron. Eng..

[7] De Smedt Q., Wannous H., Vandeborre J.P. Skeleton-based dynamic hand gesture recognition. Proceedings of the IEEE Conference on Computer Vision and Pattern Recognition Workshops.

[8] Cao Z., Simon T., Wei S.E., Sheikh Y. Realtime multi-person 2d pose estimation using part affinity fields. Proceedings of the IEEE Conference on Computer Vision and Pattern Recognition.

[9] Zhang F., Bazarevsky V., Vakunov A., Tkachenka A., Sung G., Chang C.L., Grundmann M. (2020). Mediapipe hands: On-device real-time hand tracking. arXiv.

[10] Yan S., Xiong Y., Lin D. Spatial temporal graph convolutional networks for skeleton-based action recognition. Proceedings of the AAAI Conference on Artificial Intelligence.

[11] Dosovitskiy A. (2020). An image is worth 16x16 words: Transformers for image recognition at scale. arXiv.

[12] Liu Z., Wang Y., Vaidya S., Ruehle F., Halverson J., Soljačić M., Hou T.Y., Tegmark M. (2024). Kan: Kolmogorov-arnold networks. arXiv.

[13] De Smedt Q., Wannous H., Vandeborre J.P., Guerry J., Le Saux B., Filliat D. Shrec’17 track: 3d hand gesture recognition using a depth and skeletal dataset. Proceedings of the 3DOR-10th Eurographics Workshop on 3D Object Retrieval.

[14] Boulahia S.Y., Anquetil E., Multon F., Kulpa R. Dynamic hand gesture recognition based on 3D pattern assembled trajectories. Proceedings of the 2017 Seventh International Conference on IMAGE Processing Theory, Tools and Applications (IPTA).

[15] Lamberti L., Camastra F. (2011). Real-time hand gesture recognition using a color glove. Proceedings of the Image Analysis and Processing–ICIAP 2011: 16th International Conference.

[16] Sulyman A.A., Sharef Z.T., Faraj K.H.A., Aljawaryy Z.A., Malallah F.L. (2017). REAL-TIME NUMERICAL 0-5 COUNTING BASED ON HAND-FINGER GESTURES RECOGNITION. J. Theor. Appl. Inf. Technol..

[17] Stergiopoulou E., Sgouropoulos K., Nikolaou N., Papamarkos N., Mitianoudis N. (2014). Real time hand detection in a complex background. Eng. Appl. Artif. Intell..

[18] Chen Q., Georganas N.D., Petriu E.M. Real-time vision-based hand gesture recognition using haar-like features. Proceedings of the 2007 IEEE Instrumentation & Measurement Technology Conference IMTC 2007.

[19] Zhou Y., Jiang G., Lin Y. (2016). A novel finger and hand pose estimation technique for real-time hand gesture recognition. Pattern Recognit..

[20] Ren Z., Meng J., Yuan J. Depth camera based hand gesture recognition and its applications in human-computer-interaction. Proceedings of the 2011 8th International Conference on Information, Communications & Signal Processing.

[21] Desai S. (2017). Segmentation and recognition of fingers using Microsoft Kinect. Proceedings of the International Conference on Communication and Networks: ComNet 2016.

[22] Chen Y., Zhao L., Peng X., Yuan J., Metaxas D.N. (2019). Construct dynamic graphs for hand gesture recognition via spatial-temporal attention. arXiv.

[23] Kuznetsova A., Leal-Taixé L., Rosenhahn B. Real-time sign language recognition using a consumer depth camera. Proceedings of the IEEE International Conference on Computer Vision Workshops.

[24] Marin G., Dominio F., Zanuttigh P. Hand gesture recognition with leap motion and kinect devices. Proceedings of the 2014 IEEE International Conference on Image Processing (ICIP).

[25] Nunez J.C., Cabido R., Pantrigo J.J., Montemayor A.S., Velez J.F. (2018). Convolutional neural networks and long short-term memory for skeleton-based human activity and hand gesture recognition. Pattern Recognit..

[26] Chen X., Wang G., Guo H., Zhang C., Wang H., Zhang L. (2019). Mfa-net: Motion feature augmented network for dynamic hand gesture recognition from skeletal data. Sensors.

[27] Zhang W., Lin Z., Cheng J., Ma C., Deng X., Wang H. (2020). Sta-gcn: Two-stream graph convolutional network with spatial–temporal attention for hand gesture recognition. Vis. Comput..

[28] Li M., Chen S., Chen X., Zhang Y., Wang Y., Tian Q. Actional-structural graph convolutional networks for skeleton-based action recognition. Proceedings of the IEEE/CVF Conference on Computer Vision and Pattern Recognition.

[29] Shi L., Zhang Y., Cheng J., Lu H. Two-stream adaptive graph convolutional networks for skeleton-based action recognition. Proceedings of the IEEE/CVF Conference on Computer Vision and Pattern Recognition.

[30] Li R., Wang S., Zhu F., Huang J. Adaptive graph convolutional neural networks. Proceedings of the AAAI Conference on Artificial Intelligence.

[31] Vaswani A. Attention is all you need. Proceedings of the Advances in Neural Information Processing Systems.

[32] Plizzari C., Cannici M., Matteucci M. (2021). Skeleton-based action recognition via spatial and temporal transformer networks. Comput. Vis. Image Underst..

[33] Han X., Cui Y., Chen X., Lu Y., Hu W. (2024). Spatio-Temporal Dynamic Attention Graph Convolutional Network Based on Skeleton Gesture Recognition. Electronics.

[34] Shi L., Zhang Y., Cheng J., Lu H. Decoupled spatial-temporal attention network for skeleton-based action-gesture recognition. Proceedings of the Asian Conference on Computer Vision.

[35] Li Y., Wei G., Desrosiers C., Zhou Y. (2024). Decoupled and boosted learning for skeleton-based dynamic hand gesture recognition. Pattern Recognit..

[36] Slama R., Rabah W., Wannous H. Str-gcn: Dual spatial graph convolutional network and transformer graph encoder for 3d hand gesture recognition. Proceedings of the 2023 IEEE 17th International Conference on Automatic Face and Gesture Recognition (FG).

[37] Song J.H., Kong K., Kang S.J. (2022). Dynamic hand gesture recognition using improved spatio-temporal graph convolutional network. IEEE Trans. Circuits Syst. Video Technol..

[38] Liu J., Wang X., Wang C., Gao Y., Liu M. (2023). Temporal decoupling graph convolutional network for skeleton-based gesture recognition. IEEE Trans. Multimed..

[39] Mohammed A.A., Lv J., Islam M.S., Sang Y. (2023). Multi-model ensemble gesture recognition network for high-accuracy dynamic hand gesture recognition. J. Ambient Intell. Humaniz. Comput..

[40] AQ Mohammed A., Gao Y., Ji Z., Lv J., Sajjatul Islam M., Sang Y. Automatic 3D Skeleton-based Dynamic Hand Gesture Recognition Using Multi-Layer Convolutional LSTM. Proceedings of the 7th International Conference on Robotics and Artificial Intelligence.

